# 

**Published:** 1889-07-06

**Authors:** 


SATURDAY, JULY 6th, 1889.

**A Public Medical Service for England** - 207
**The School of Medicine for Women** - 208
**The British Nurses' Association: An Argument and a Warning.** By A. Ernest Sansom, M.D. - 209
**Within the Wards.**—About Chemical Formulæ - 210
**The Doctor's Pulpit.**—In Nature's School - 211
**"The Scarlet Letter"** - 212
**Jenner and Vaccination** - 213
**Notes and News** - 214
**Everybody's Page** - 216
**Annotations.**—Heat, Heads, Hats!—The Vaccination Commission sitting in Private—The Horse's Turn - 217
**The Hospital Workshop.**—No. IV. The Throat Department at St. Mary's Hospital - 218
**Her Heart's Mistake.** By E. D. Cumming. - 219
**Summer Diarrhœa and Cholera.** By Sir William Moore, K.C.I.E. - 221
**Britain's Native Resources.**—Flitwick Water - 221
**Amusements and Relaxation** - 222
**Scraps and Gleanings** - 222

**EXTRA SUPPLEMENT—THE NURSING MIRROR.**

**En Passant.**—In Season and Out of Season—Registration—An Important Meeting—The New Hospital for Women—Women as Guardians—Imbeciles - li

**Notes from Australia** - lii

**Dare We Utter Our Opinions** - liii

**Children's Country Holidays Fund** - liv

**Everybody's Opinion.**—Ladies as Poor Law Guardians—Infirmary Nursing—Convalescent Home for Hip Cases - liii

**Appointments** - liv

**Vacancies** - liv

**Notes and Queries** - liv

**Death in Our Ranks** - liv

